# Prospective association between sleep duration and cognitive impairment: Findings from the China Health and Retirement Longitudinal Study (CHARLS)

**DOI:** 10.3389/fmed.2022.971510

**Published:** 2022-09-06

**Authors:** Wenhua Liu, Qingsong Wu, Minghuan Wang, Peng Wang, Na Shen

**Affiliations:** ^1^Clinical Research Center, Tongji Hospital, Tongji Medical College, Huazhong University of Science and Technology, Wuhan, China; ^2^Department of Scientific Research Management, Union Hospital, Tongji Medical College, Huazhong University of Science and Technology, Wuhan, China; ^3^Department of Neurology, Tongji Hospital, Tongji Medical College, Huazhong University of Science and Technology, Wuhan, China; ^4^Institute and Department of Infectious Disease, Tongji Hospital, Tongji Medical College, Huazhong University of Science and Technology, Wuhan, China; ^5^Department of Laboratory Medicine, Tongji Hospital, Tongji Medical College, Huazhong University of Science and Technology, Wuhan, China

**Keywords:** sleep duration, napping, cognitive impairment, risk, changes in sleep duration

## Abstract

**Objective:**

The association between sleep duration and cognition are inconclusive. Our study aimed to comprehensively investigate the effects of sleep duration on the risk of cognitive impairment in the middle-aged and older Chinese population.

**Methods:**

We used the longitudinal cohort data from waves 1–4 (2011–2018) of the China Health and Retirement Longitudinal Study (CHARLS). Self-reported exposures included total sleep duration, nocturnal sleep duration, post-lunch napping, and changes in sleep duration over time according to face-to-face interviews. Cognitive function was assessed by a Chinese version of the Modified Mini-Mental State Examination (MMSE).

**Results:**

A total of 7,342 eligible participants were included. The mean age was 61.5 ± 6.5 years, and 48.9% (3,588/7,342) were male. We identified a U-shaped association of total sleep duration as well as nocturnal sleep duration with the risk of cognitive impairment. People with 7–8 h of total sleep duration and 6–7 h of nocturnal sleep had the lowest risk of cognitive impairment. Further results showed that post-lunch napping within 2 h was beneficial to cognition and 60 min was optimal. Moreover, analyses of changes in sleep duration further supported that sleeping less or more was harmful to cognition. Notably, those “excessive-change” sleepers (from ≤6 to ≥9 h, or from ≥9 to ≤6 h) had more risks.

**Conclusions:**

Keeping 7–8 h per day was related to the lowest risk of cognitive impairment in midlife and late life, and an optimal post-lunch napping was 60 min for these stable sleepers. Especially, excessive changes in sleep duration over time led to poorer cognition. Our work highlights the importance of optimal sleep habits to cognitive function. The self-reported sleep measures limited our findings, and further studies are needed for verification.

## Introduction

As a leading cause of disability for older adults, dementia is becoming a public health problem. The number of people with dementia is about 50 million worldwide and will increase to 152 million by 2050 (1). In China, there are 10 million people with dementia, accounting for 25% of the patients with dementia in the world (2). So far there is no effective treatment for dementia and thus prevention strategy has become a top priority. Cognitive impairment is a cardinal feature during the long preclinical period of dementia. Therefore, to identify modifiable risk factors of cognitive impairment is essential for dementia prevention (3).

Several risk factors including age, sex and marital status are confirmed in the development of cognitive impairment or dementia (4–7). Nowadays, an increasing number of studies have reported a strong association between sleep and cognition. Experimental studies found the effects of sleep and circadian rhythm on cognitive impairment (8, 9). Population studies also supported the role of sleep duration in cognitive function. It is estimated that about 15% of Alzheimer’s disease in the population may be due to sleep problems (10). One study showed that both self-reported short and long sleep durations were risk factors for cognitive aging (11). Two dose-response meta-analyses showed a U-shaped association between total sleep duration and the risk of cognitive disorders (12, 13). Ma et al. identified an inverted U-shaped association of nocturnal sleep duration with global cognitive decline (14). Napping is another important sleep behavior. It is estimated that 22–69% of older adults have habitual daytime napping in the world (15–17). Previous studies explored the short- or long-term effects of daytime napping on cognition, but findings are inconsistent (15, 18–20). In addition, the association between changes in sleep duration and cognition is attracting more and more attention. However, some cohort studies (21, 22) observed that both increased and decreased sleep duration were related to poor cognition, whereas other studies (23–25) did not support this conclusion. Two recent studies exhibited an association of excessive changes in sleep duration with lower cognitive function (26, 27). Although sleep duration is associated with cognition, few studies focus on the effects of different types of sleep duration.

Previous studies often focused on the effect of one behavior of sleep on cognition, and possibly limited by sample size or methodology. Even in the large cohort studies, results are not fully consistent and need more verification. Therefore, we performed this study to comprehensively examine the association between different types of sleep behaviors and cognitive function, using the data from a large population-based prospective cohort, the China Health and Retirement Longitudinal Study (CHARLS) (28). Based on data from four waves of CHARLS, we aimed to explore the following issues: (1) the dose-response association of total and nocturnal sleep duration with the risk of cognitive impairment; (2) effects of nocturnal sleep and napping on cognitive function; and (3) longitudinal associations between changes in total sleep duration and cognitive impairment risk.

## Materials and methods

### Study population

This study used the publicly available data from the CHARLS^1^ (28). CHARLS is an ongoing national longitudinal survey, covering 450 urban and rural areas from 28 provinces of China. Demographic characteristics, health outcomes and family information were assessed in this cohort. At baseline, a total of 17,708 participants were recruited. Ethical approval for the data collection of the CHARLS was obtained from the Institutional Review board of Peking University (IRB00001052-11015). All participants provided written informed consents during the investigation. We collected the data four waves of CHARLS, including 2011 wave 1 (baseline), 2013 wave 2, 2015 wave 3, and 2018 wave 4 (28).

The inclusion criteria in this study were as follows: (1) participants at baseline attended the Modified Mini-Mental State Examination (MMSE) in 2018 wave 4; and (2) age ≥60 years old at 2018 wave 4. Hence a total of 9,106 subjects were included. Then the following exclusion criteria were applied: (1) reporting unusual values of sleep duration (<3 or >18 h per night) (*n* = 392); (2) missing data of sleep duration at baseline or MMSE scores (*n* = 1,217); and (3) self-reported diagnoses of mental illness or neurological disease (*n* = 155). Finally, we included 7,342 eligible participants for analyses (Supplementary Figure 1).

### Measurements

#### Cognitive assessment

Cognitive function was measured using the Chinese version of the MMSE test. It is a good tool to evaluate cognitive function, which has good validity and reliability in Alzheimer’s disease patients as well as in the general population (29–31). MMSE items include memory, orientation, language, and attention and computation, with a total score of 30. The lower score indicates the poorer cognitive function. Cutoffs were described as 18 for illiterate individuals, 21 for participants with ≤6 years of education, and 25 for participants with >6 years of education (31). Cognitive impairment was considered as participants who had the MMSE scores lower than cutoffs based on the years of education above.

#### Sleep variables

Four sleep variables were investigated: nocturnal sleep duration, post-lunch napping, total sleep duration, and changes in sleep duration over time. The nocturnal sleep duration and post-lunch napping were collected from two questions at each wave by face-to-face interviews: (1) “During the past month, how many hours of actual sleep did you get at night?” (2) “During the past month, how long (minutes) did you take a nap after lunch?” Total sleep duration was the sum of nocturnal sleep and post-lunch napping. When describing the characteristics of participants, both of total sleep duration and nocturnal sleep duration at baseline were divided into six groups: ≤5, 6, 7, 8, 9, and ≥10 h. Post-lunch napping was divided into four groups: <30, 30–90, and >90 min. The cut points for sleep durations and post-lunch napping were according to previous studies (15, 32). Changes in sleep duration over time were evaluated from baseline to 2013 wave 2, and from baseline to 2015 wave 3.

#### Covariates

Demographic characteristics and other potential confounders were considered as covariates in this study. They included age, sex, marital status, education level, socioeconomic groups, urbanization, smoking, drinking, Activities of daily living (ADL), social activity scores, and chronic disease comorbidity status.

Marital status was divided into two groups: married and others (divorced, widowed, or never married). Education was categorized as illiterate, primary school (≤6 years of education), and secondary school or higher (>6 years of education). The annual household consumption spending per capita was used as a proxy for socioeconomic status, as described by Zhao et al. (33). We considered four socioeconomic groups based on the quartiles of annual household consumption spending per capita stratified by provinces. Smoking status was categorized as current smokers and non-smokers (e.g., never smokers or ex-smokers). Drinking status was categorized as current drinkers and non-drinkers (e.g., never drinkers or ex-drinkers). Chronic disease comorbidity referred to 11 kinds of non-communicable diseases, including self-reported diagnosed hypertension, diabetes, heart disease, chronic lung disease, digestive disease, live disease, kidney disease, dyslipidemia, stroke, cancer, and arthritis. In addition, participants who did not report hypertension but have systolic blood pressure ≥140 mmHg or diastolic blood pressure ≥90 mmHg were also considered as hypertension. Participants who did not report diabetes but have glycated hemoglobin ≥6.5% were also considered as diabetes. According to the number of these diseases, disease status was defined as three groups: none, one or two, and three or more. ADL included six items: eating, dressing, bathing, transferring, continence, and using the toilet. Participants were classified as two groups: without difficulty (not needing assistance for all items of ADL), and with difficulty (needing assistance for any item of ADL). Based on the number of social activities, social scores were divided into three groups: 0, 1, and ≥2. Social activities were as follows: (1) communicating with friends; (2) playing Ma-jong, chess, or cards, or going to community club; (3) volunteering to help family, friends, or neighbors who do not live with you; (4) going to a sport, social or other kinds of club; (5) taking part in a community-related organization; (6) doing voluntary work for a charity; (7) volunteering to take care of a sick or disable person who do not live with you; (8) attending an educational or training course; (9) investing in stock market; and (10) using the internet. The frequency of the activities above should be daily or almost every week in the last month.

### Statistical analyses

Baseline characteristics were shown as mean ± standard deviation (SD) or frequency/percentages. Group differences were examined using Student’s *t*-test or Kruskal–Wallis test for continues variables and Pearson’s χ^2^ test for categorical variables. Multiple logistic regression models were used to investigate the associations between baseline sleep variables and the risk of cognitive impairment after adjustment of covariates. In the characteristics description, sleep variables were roughly shown as categorical data. For deeply explore their association with the risk of cognitive impairment, they were treated as continuous data in further restricted cubic spline functions. We used the restricted cubic spline function with 3 knots of “6, 8, and 10 h” in the multiple logistic regression model to explore the dose-response association between total sleep duration at baseline and cognitive impairment risk in the whole and subgroup participants. Then we used the knots of “5, 7, and 9 h” to explore the dose-response association between nocturnal sleep duration at baseline and cognitive impairment risk. Moreover, we explored the effects of post-lunch napping on cognition based on different nocturnal sleep durations using a multiple logistic regression with restricted cubic spline function (“0, 30, and 90 min” as knots). For analyses of changes in sleep duration, we classified participants into five groups according to total sleep duration between baseline and wave 2, and between baseline and wave 3 as follows: stable moderate sleepers (always 7–8 h), moderate-to-unhealthy sleepers (from 7–8 h to ≤6 or ≥9 h), unhealthy-to-moderate sleepers (from ≤6 or ≥9 h to 7–8 h), short or long sleepers (always ≤6 or ≥9 h), and “excessive-change” sleepers (from ≤6 to ≥9 h, or from ≥9 to ≤6 h). The changes in total sleep duration from baseline to Wave 2, and from baseline to Wave 3 were explored separately. Odds ratio (OR) and 95% confidence interval (CI) were used to measure the association strength of risk. Statistical analyses were conducted by Stata 15.0 (College Station, TX, United States). A two-sided *P* value <0.05 was considered statistically significant.

## Results

### Sample characteristics

A total of 7,342 eligible participants at baseline were included for analyses. The mean age was 61.5 ± 6.5 years, and 48.9% (3,588/7,342) were male. Of these, 3,675 (50.0%) participants had cognitive impairment based on the MMSE scores in the 2018 wave 4. Nocturnal sleep duration, post-lunch napping and total sleep duration demonstrated significant associations with cognitive impairment. Among other baseline factors, older age, female, not married, smoking, and ADL with difficulty increased the risk of cognitive impairment. Living in urban, higher level of education, higher socioeconomic status, and more social scores played a protective role of cognitive function. These cognition-related covariates were adjusted in the following analyses dealing with sleep variables. Details are shown in Table 1 and Supplementary Table 1.

**TABLE 1:** Baseline characteristics of the participants according to the cognitive function.

Baseline characteristics	Number	Cognitive impairment group (%)	Cognitive normal group (%)	*t*/χ ^2^	*P* value
Total	7,342	3,675 (50.0)	3,667 (50.0)		
Age (years)	7,342	62.2 ±7.0	60.8 ±5.8	9.20	<0.001
**Sex**					
Male	3,588	1,540 (42.9)	2,048 (57.1)	142.84	<0.001
Female	3,754	2,135 (56.9)	1,619 (43.1)		
**Urbanization**					
Rural	4,666	2,587 (55.4)	2,079 (44.6)	148.72	<0.001
Urban	2,676	1,088 (40.7)	1,588 (59.3)		
**Marital status**					
Married	6,438	3,104 (48.2)	3,334 (51.8)	70.87	<0.001
Other	904	571 (63.2)	333 (36.8)		
**Education level**					
Illiterate	2,220	1,567 (70.6)	653 (29.4)	557.74	<0.001
Primary school	4,427	1,877 (42.4)	2,550 (57.6)		
Secondary school or higher	692	229 (33.1)	463 (66.9)		
**Socioeconomic status**					
Quartile 1	1,844	1,063 (57.7)	781 (42.4)	111.90	<0.001
Quartile 2	1,831	975 (53.3)	856 (46.8)		
Quartile 3	1,826	883 (48.4)	943 (51.6)		
Quartile 4	1,787	731 (40.9)	1,056 (59.1)		
**Smoking status**					
Non-smokers	4,995	2,585 (51.8)	2,410 (48.3)	18.01	<0.001
Current smokers	2,347	1,090 (46.4)	1,257 (53.6)		
**Drinking status**					
Non- drinkers	5,493	2,831 (51.5)	2,662 (48.5)	19.21	<0.001
Current drinkers	1,849	844 (45.7)	1,005 (54.4)		
**Chronic disease comorbidity status**					
0	2,253	1,133 (50.3)	1,120 (49.7)	0.61	0.736
1∼2	3,811	1,915 (50.3)	1,896 (49.8)		
>2	1,278	627 (49.1)	651 (50.9)		
**ADL**					
With difficulty	1,145	681 (59.5)	464 (40.5)	48.17	<0.001
Without difficulty	6,197	2,994 (48.3)	3,203 (51.7)		
**Social scores**					
0	4,738	2,537 (53.6)	2,201 (46.5)	89.78	<0.001
1	1,984	921 (46.4)	1,063 (53.6)		
2	620	217 (35)	403 (65)		
**Nocturnal sleep duration (h)**					
≤5	2,124	1,143 (53.8)	981 (46.2)	62.14	<0.001
6	1,607	752 (46.8)	855 (53.2)		
7	1,433	632 (44.1)	801 (55.9)		
8	1,587	794 (50)	793 (50)		
9	299	181 (60.5)	118 (39.5)		
≥10	292	173 (59.3)	119 (40.8)		
**Post-lunch napping (min)**					
0	3,460	1,859 (53.7)	1,601 (46.3)	43.52	<0.001
1∼29	1,216	591 (48.6)	625 (51.4)		
30∼90	1,860	826 (44.4)	1,034 (55.6)		
>90	806	399 (49.5)	407 (50.5)		
**Total sleep duration (h)**					
≤5	1,625	908 (55.9)	717 (44.1)	47.67	<0.001
6	1,259	620 (49.3)	639 (50.8)		
7	1,417	649 (45.8)	768 (54.2)		
8	1,490	687 (46.1)	803 (53.9)		
9	832	419 (50.4)	413 (49.6)		
≥10	719	392 (54.5)	327 (45.5)		

Data was shown as mean ± standard deviation (SD) for continues variables or frequency (percentage) for categorical variables. Three participants had missing values for education level, and 54 had missing values for socioeconomic status.

### Association of total sleep duration with risk of cognitive impairment

**FIGURE 1:**
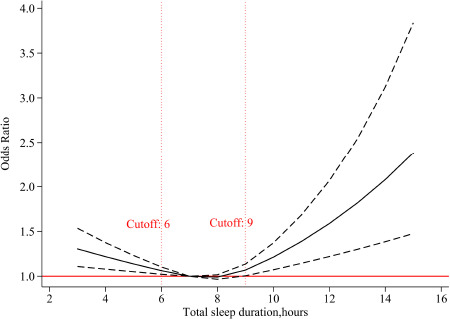
The adjusted dose-response association between total sleep duration at baseline and risk of cognitive impairment. Total sleep duration at baseline was modeled *via* a restricted cubic spline function with knots of 6, 8, 10, and 7 h was as the reference value.

A U-shaped association was observed between total sleep duration at baseline and risk of cognitive impairment, based on the restricted cubic spline logistic regression model. As shown in Figure 1, the moderate sleep duration was 7–8 h per day, and sleep less (≤6 h) or more (≥9 h) significantly increased the risk of cognitive impairment. Similar U-shaped patterns were also observed in the subgroup analyses by sex and age, and females and younger age seemed to be more affected by sleep duration (Supplementary Figure 2).

### Association of nocturnal sleep duration and post-lunch napping with risk of cognitive impairment

**FIGURE 2:**
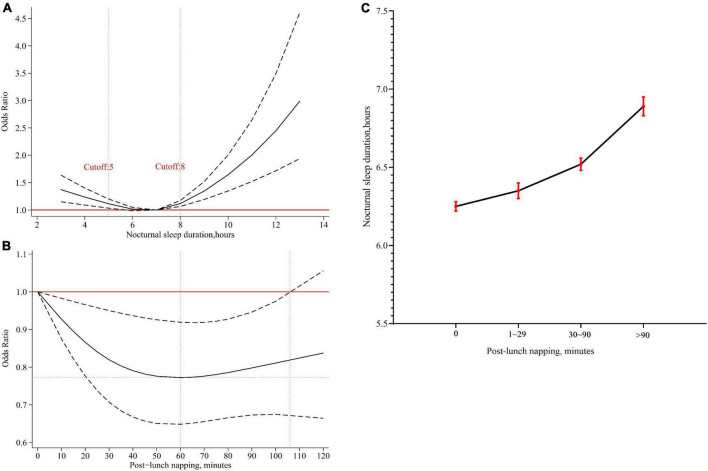
The effects of nocturnal sleep duration or post-lunch napping at baseline on cognition. **(A)** The adjusted dose-response association between nocturnal sleep duration at baseline and risk of cognitive impairment. Nocturnal sleep duration at baseline was modeled *via* a restricted cubic spline logistic regression model with knots of 5, 7, 9, and 7 h was as the reference value. **(B)** The adjusted dose-response association between post-lunch napping at baseline and risk of cognitive impairment among participants sleeping 6–7 h at night. Post-lunch napping at baseline was modeled *via* a restricted cubic spline logistic regression model with knots of 0, 30, and 90 min, and non-napping was as the reference value. **(C)** Association between post-lunch napping and nocturnal sleep duration. The black dots represented mean nocturnal sleep duration, and the red error bars represented 95% confidence intervals.

As shown in Figure 2A, a U-shaped association suggested that 6–7 h of nocturnal sleep duration at baseline had the lowest risk of cognitive impairment. Short (≤5 h) or long (≥8 h) nocturnal sleep duration was harmful to cognitive function.

Based on these results, the role of post-lunch napping at baseline was further explored. We identified that post-lunch napping significantly decreased the risk of cognitive impairment in participants sleeping 6–7 h at night (OR = 0.77, 95% CI = 0.66–0.90, *P* = 0.001, Table 2). Dose-response analysis showed that less than 110 min of post-lunch napping helped decrease the risk of cognitive impairment, and 60 min was likely to be the best duration (Figure 2B). However, the significant effects of post-lunch napping on cognition were not found among participants with short (≤5 h) or long (≥8 h) nocturnal sleep duration (Table 2).

**TABLE 2:** The association between post-lunch napping at baseline and risk of cognitive impairment stratified by nocturnal sleep duration.

Nocturnal sleep duration (h)	Post-lunch napping	Number of participants (%)	OR (95% CI)	*P* value
≤5	No	1,134 (53.4)	Reference	
	Yes	990 (46.6)	1.12 (0.93, 1.35)	0.247
6∼7	No	1,378 (45.3)	Reference	
	Yes	1,662 (54.7)	0.77 (0.66, 0.90)	0.001
≥8	No	948 (43.5)	Reference	
	Yes	1,230 (56.5)	0.85 (0.71, 1.02)	0.085

Multiple logistic regression models were used to investigate the associations between post-lunch napping at baseline and the risk of cognitive impairment stratified by nocturnal sleep duration with adjustment of potential confounders: age, sex, marital status, education level, socio-economic status, urbanization, smoking, drinking, ADL, social activity score, and chronic disease comorbidity status.

Further analysis revealed that post-lunch napping was significantly related to nocturnal sleep duration (All *P* < 0.001, Supplementary Table 2). Overall, participants who reported less nocturnal sleep duration tended to have less napping, and vice versa (Figure 2C).

### Association of changes in total sleep duration with risk of cognitive impairment

For changes in total sleep duration over time, we analyzed the data from baseline to 2013 wave 2, and from baseline to 2015 wave 3 (Table 3). Overall, compared with stable moderate sleepers (always 7–8 h), other sleepers showed an increased risk of cognitive impairment or similar tendency. Compared with stable moderate sleepers (always 7–8 h), short or long sleepers (always ≤6 or ≥9 h) had an increased risk of cognitive impairment (from baseline to wave 2: OR = 1.22, 95% CI = 1.04–1.42, *P* = 0.012; from baseline to wave 3: OR = 1.19, 95% CI = 1.02–1.38, *P* = 0.027). Notably, those “excessive-change” sleepers (from ≤6 to ≥9 h, or from ≥9 to ≤6 h) likely had more risks (from baseline to wave 2: OR = 1.63, 95% CI = 1.30–2.03, *P* < 0.001; from baseline to wave 3: OR = 1.58, 95% CI = 1.27–1.96, *P* < 0.001).

**TABLE 3:** The association between change of total sleep duration and risk of cognitive impairment.

Changes in total sleep duration*	From baseline to 2013 Wave			From baseline to 2015 Wave		
	Number of participants (%)	OR (95% CI)	*P* value	Number of participants (%)	OR (95% CI)	*P* value
Stable moderate sleepers	1,220 (19.2)	Reference		1,183 (18.1)	Reference	
Moderate-to-Unhealthy sleepers	1,315 (20.7)	1.19 (1.01, 1.41)	0.039	1,434 (21.9)	1.05 (0.89, 1.24)	0.538
Unhealthy-to-Moderate sleepers	1,297 (20.4)	1.24 (1.05, 1.47)	0.010	1,316 (20.1)	1.12 (0.95,1.32)	0.193
Short or Long sleepers	2,011 (31.6)	1.22 (1.04, 1.42)	0.012	2,063 (31.1)	1.19 (1.02, 1.38)	0.027
“Excessive-change” sleepers	512 (8.1)	1.63 (1.30, 2.03)	<0.001	571 (8.8)	1.58 (1.27, 1.96)	<0.001

*Stable moderate sleepers: always 7–8 h, Moderate-to-Unhealthy sleepers: from 7–8 h to ≤6 or ≥9 h, Unhealthy-to-Moderate sleepers: from ≤6 or ≥9 h to 7–8 h, Short sleepers: always ≤6 h, Long sleepers: always ≥9 h, “Excessive-change” sleepers: from ≤6 to ≥9 h, or from ≥9 to ≤6 h. Multiple logistic regression models were used to investigate the associations between change of total sleep duration and the risk of cognitive impairment with adjustment of potential confounders: age, sex, marital status, education level, socio-economic status, urbanization, smoking, drinking, ADL, social activity score, and chronic disease comorbidity status.

## Discussion

In this nationally representative community-based cohort including 7,342 middle-aged and older subjects, we identified a significant U-shaped association between total sleep duration and cognitive function. A similar U-shaped pattern was also observed in nocturnal sleep. We also found that 7–8 h of total sleep duration and 6–7 h of nocturnal sleep had the lowest risk of poor cognition. Post-lunch napping within 2 h could decrease the risk of cognitive impairment, and 60 min might be an optimal choice. Longitudinal analysis highlighted that excessive changes in sleep duration over time had more risk of cognitive impairment than sleeping less or more. The unhealthy sleep patterns over time were significantly modified by some factors including social activities.

Previous cohort studies have investigated the association between sleep duration and cognitive disorders but had inconsistent results, such as the US Nurses’ Health Study by Tworoge et al. (34), the Finnish Twin Cohort by Virta et al. (35), and Screening Across the Lifespan Twin study by Bokenberger et al. (36). Possible reasons included not large sample size (*n* < 2,000), short follow-up period (<3 years), or weak in representativeness (e.g., only one sex or twin study). Several meta-analyses were performed and found a U-shaped pattern of sleep duration in cognition, but they did not distinguish total sleep duration from nocturnal sleep duration (12, 13). Recently, Ma, et al. combined data from the English Longitudinal Study of Ageing (ELSA) and CHARLS, also reported a U-shaped association between nocturnal sleep duration and cognitive function (14). Consistently, we identified a U-shaped association of total sleep duration as well as nocturnal sleep duration with the risk of cognitive impairment. Compared with the studies by Ma et al. (14), we evaluated more sleep variables based on the data from more waves of survey and information from a new comprehensive assessment tool of cognitive outcome in the CHARLS. Moreover, we recommended 7–8 h for total sleep or 6–7 h for nocturnal sleep per day, because such sleep durations had the lowest risk of cognitive impairment. A recent study with sleep duration measured by polysomnography also supported our results (37). Lucey et al. investigated the relationship between cognitive performance and sleep duration measured by the single-channel EEG device, and showed that individuals with sleeping less than 4.5 or more than 6.5 h at night had declined cognitive scores (37). Because the sleep time is about one hour shorter measured by EEG than by self-reported, the results corresponded to 5.5–7.5 h per night of self-reported sleep time to have the lowest risk (34, 37). Although the biological mechanisms of extreme sleep durations on cognition is still unclear, putative pathways included circadian dysfunction (38), increased accumulation of amyloid-β in the brain (39), and elevate levels of systemic inflammatory markers (e.g., C-reactive protein, interleukin-6) (40–42). In addition, we observed that sleep seemed to affect females more than males on cognition, possibly due to the hormonal and biological differences between the sexes (43). We also observed that the effects of total sleep duration on the risk of cognitive impairment decreased with age, suggesting that it is better to perform sleeping intervention as early as possible.

Daytime napping is prevalent in Asians. Previous studies have reported that moderate daytime napping could improve cognitive performance in the ageing people (15, 44, 45). Daytime napping can regulate the inflammatory response and reduce fatigue in the day (46), but excessive napping may develop sedentary habit and be considered a risk marker of amyloid-β accumulation (47). Consistently, our work also supported the benefits of post-lunch napping on cognition, and had more details in moderate napping time. We found that in the participants with 6–7 h of nocturnal sleep, post-lunch napping within 2 h led to better cognition, and the optimal napping time was 60 min. Further results showed that short nappers often slept less at night, and vice versa. That might imply that different types of nocturnal sleep have certain napping habits, which possibly play different roles on cognition. Supportive evidence was that a national survey determined the beneficial effect of long nap durations on cognitive performance only in elderly adults with a morning chronotype, rather than in those with other chronotypes (48). Another explanation was that the nocturnal sleep habit probably masked the beneficial effect of napping on cognition for participants who slept less (≤5 h) or more (≥8 h) at night.

Recently, some studies have focused on associations of changes in sleep duration and cognitive function, but results are conflicting (21–25). Our work demonstrated that short or long sleepers (always ≤6 or ≥9 h) had more risk of cognitive impairment than no-change moderate sleepers (always 7–8 h). This result was in line with the recommendation by the National Sleep Foundation that 7–8 h of sleep is good for the elderly (49). Moreover, we further found that the highest risk of cognitive impairment was in those who had excessive changes in sleep duration (from ≤6 to ≥9 h, or from ≥9 to ≤6 h). Supportive evidence can be found in the latest studies (26, 27, 50). Hua et al. used the nocturnal sleep data of three waves of CHARLS (2011–2015) and found that an increased or decreased change of ≥2 h in sleep duration correlated with poorer cognition (26). Wu et al. reported that substantial changes in sleep duration (e.g., from ≤5 to ≥9 h) was associated with a higher risk of cognitive impairment (27). Even for the mild cognitive impairment, keeping stable sleep duration in a normal range is essential for better cognition (50). A possible explanation that shifts of sleep duration may increase the levels of inflammatory markers and contribute to the decline cognition (41). Other interpretations included amyloid-β deposition and circadian dysfunction (38, 39), but exact mechanisms are not very clear and need more investigations.

Actually, abnormal sleep durations are common health problems rather than simple behaviors among the middle-aged and older adults. For example, excessive daytime sleepiness (EDS) can cause falls, depression and cognitive impairment, and was estimated to occur in about 20% of the older people (51). A recent study investigated the association between malnutrition and excessive daytime sleepiness (EDS) in patients with and without dementia, and found that malnutrition, dysphagia, and vitamin D deficiency were significantly related to EDS (52). Interestingly, nutritional status was also reported to be closely associated with insomnia or insomnia severity in older adults (53). These issues should be considered in the association between sleep behaviors and cognitive function in the future.

This study has major strengths in a large and nationally representative sample, many waves of follow-ups, multiple adjustment of potential confounders, and comprehensive analyses of sleep behaviors. However, several limitations should also be acknowledged. First, the sleep variables were collected by self-reported data rather than objective measurements, which might be influenced by recall bias and lead to exposure misclassification. Second, although we adjusted for a number of potential confounders, residual confounding still possibly occurred due to unmeasured covariates, including other sleep disturbances (e.g., parasomnias), napping frequency and nutrition conditions. Influences of these factors should be considered in future studies. Third, MMSE is a good screening tool to identify dementia, but it is insensitive to detect mild cognitive impairment or early-stage dementia, which could not replace the clinical diagnosis. Fourth, the cognitive assessment based on MMSE in this study was only performed in the latest follow-up. Although we excluded participants with self-reported diagnoses of mental illness or neurological disease, we could not obtain the object measurements of cognitive function at baseline and also not capture the cognition decline over time. At last, cognitive impairment has many types (e.g., Alzheimer’s disease and dementia with Lewy bodies) (54), which may be associated with types of sleep problems and subgroup analyses may provide more information for clinicians. Due to insufficient information collected in this study, we could not perform such analyses. Based on these limitations above, our findings should be cautiously interpreted and more detailed studies are still warranted in the future.

In summary, keeping 7–8 h per day in midlife and late life was related to the lowest risk of cognitive impairment, and an optimal post-lunch napping was 60 min for these stable sleepers. Moreover, excessive changes in sleep duration could result in poorer cognition. To date, “sleep” has not mentioned as a lifestyle intervention for dementia, and three large multi-domain trials (FINGER, MAPT and PreDIVA) regarding interventions to prevent cognitive disorders did not include sleep duration (55). Our findings provide new evidence for lifestyle intervention studies on cognitive impairment in the future.

## Data availability statement

The datasets presented in this study can be found in online repositories. The names of the repository/repositories and accession number(s) can be found in the article/Supplementary material.

## Ethics statement

Ethical approval for the data collection of the CHARLS was obtained from the Institutional Review board of Peking University. The patients/participants provided their written informed consent to participate in this study.

## Author contributions

NS conceived, designed, and supervised the study. WL and QW performed the formal analyses and wrote the first draft of the manuscript. MW and PW collected and reviewed the data. All authors critically revised successive drafts of the manuscript and approved the final version.

## Footnotes

1
http://charls.pku.edu.cn
