# Supplementary material for: Prospective association between sleep duration and cognitive impairment: Findings from the China Health and Retirement Longitudinal Study (CHARLS)
Source: Front Med (Lausanne). 2022 Sep 6;9:971510. doi: 10.3389/fmed.2022.971510 (PMC9485441; doi:10.3389/fmed.2022.971510)
Supplement: Supplementary file 1 [file Data_Sheet_1.pdf]

# Supplementary Appendix

**This appendix has been provided by authors to give readers additional information about their work.**

**Supplement to: Prospective Association between Sleep Duration and Cognitive Impairment: Findings from the China Health and Retirement Longitudinal Study (CHARLS)**

## Contents

**Figure S1**. Flow chart of participant selection ........................................................................ 3

**Figure S2**. The adjusted dose-response association between total sleep duration at baseline and risk of cognitive impairment stratified by sex (A) and age (B) ......................................... 4

**Table S1**. The association between baseline covariates and risk of cognitive impairment ...... 5

**Table S2**. Association between nocturnal sleep duration and post-lunch napping .................. 6

**Figure S1. Flow chart of participant selection**

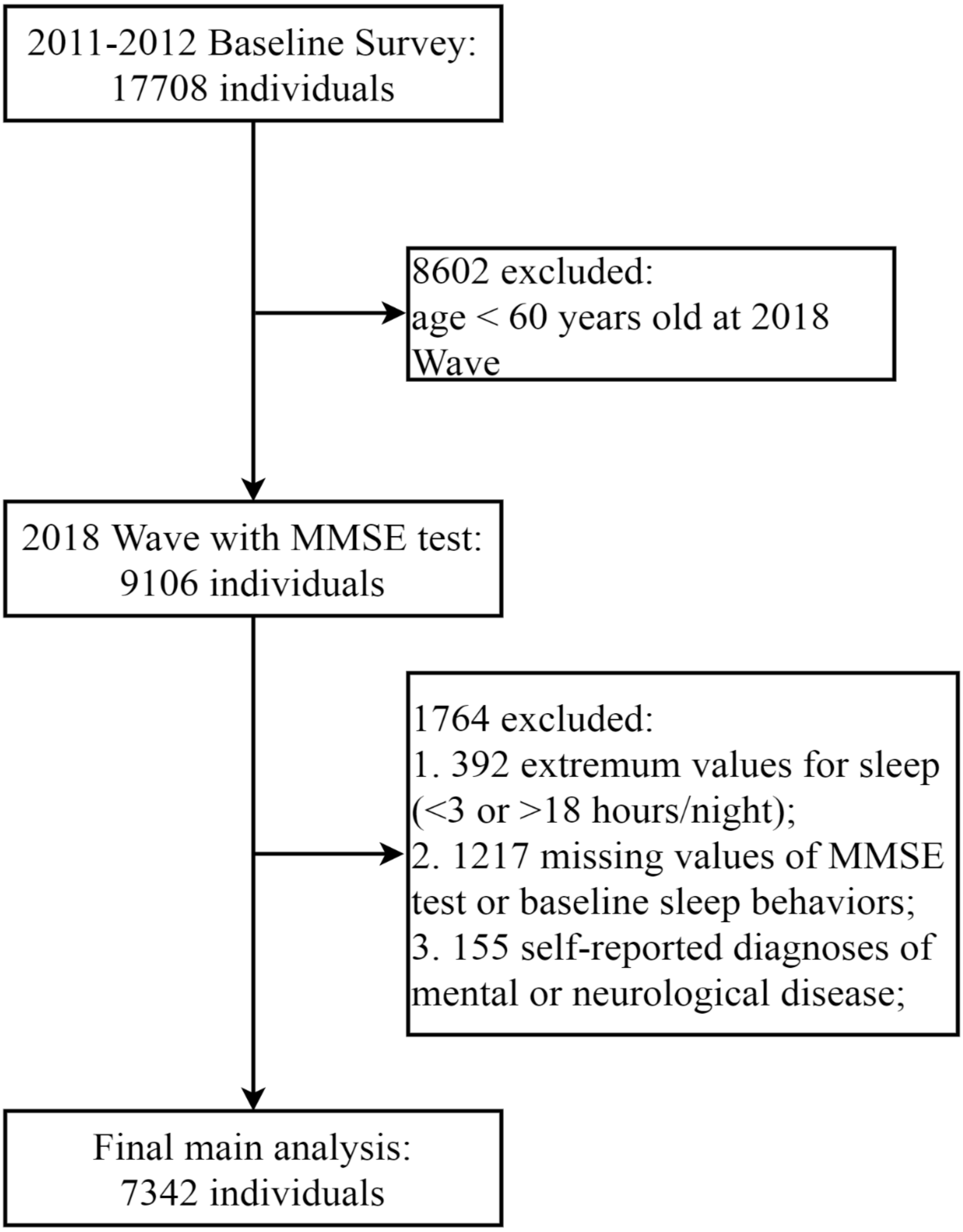


**Flow chart of participant selection, a longitudinal study of sleep duration with risk of cognitive impairment in the CHARLS, 2011-2018.**

**Figure S2. The adjusted dose-response association between total sleep duration at baseline and risk of cogniti**ve impairment **stratified by sex (A) and age (B)**

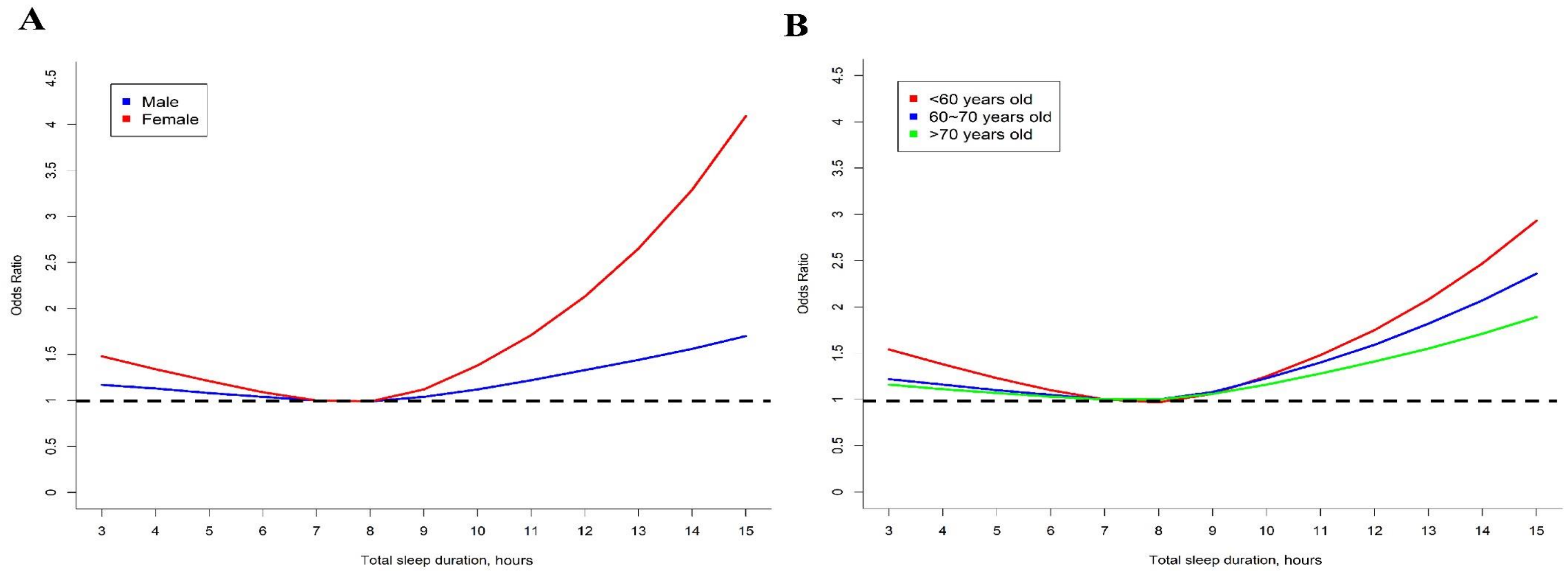


**Total sleep duration at baseline was modeled via a restricted cubic spline function with knots of 6, 8, 10 hours, and 7 hours was as the reference value for each subgroup.**

**Table S1. The association between baseline covariates and risk of cognitive impairment**

| Baseline characteristics | | OR(95%CI) | P value |
|---|---|---|---|
| **Age** | **<65 years** | Reference | |
| | **≥65 years** | 1.48(1.32, 1.66) | <0.001 |
| **Sex** | **Male** | Reference | |
| | **Female** | 1.45(1.28, 1.65) | <0.001 |
| **Urbanization** | **Rural** | Reference | |
| | **Urban** | 0.7(0.63, 0.78) | <0.001 |
| **Marital status** | **Married** | Reference | |
| | **Other** | 1.42(1.22, 1.66) | <0.001 |
| **Education level** | **Illiterate** | Reference | |
| | **Primary school** | 0.39(0.35, 0.44) | <0.001 |
| | **Secondary school or higher** | 0.35(0.29, 0.43) | <0.001 |
| **Socioeconomic status** | **Quartile 1** | Reference | |
| | **Quartile 2** | 0.9(0.79, 1.04) | 0.148 |
| | **Quartile 3** | 0.83(0.73, 0.96) | 0.009 |
| | **Quartile 4** | 0.7(0.61, 0.81) | <0.001 |
| **Smoking status** | **Non-smokers** | Reference | |
| | **Current smokers** | 1.17(1.03, 1.33) | 0.015 |
| **Drinking status** | **Non-drinkers** | Reference | |
| | **Current drinkers** | 1.06(0.93, 1.2) | 0.391 |
| **Chronic Disease Comorbidity status** | **0** | Reference | |
| | **1~2** | 0.99(0.89, 1.11) | 0.869 |
| | **>2** | 0.91(0.78, 1.05) | 0.197 |
| **ADL** | **Without difficulty** | Reference | |
| | **With difficulty** | 1.25(1.08, 1.43) | 0.002 |
| **Social scores** | **0** | Reference | |
| | **1** | 0.77(0.69, 0.86) | <0.001 |
| | **2** | 0.63(0.52, 0.75) | <0.001 |

Multiple logistic regression models were used to investigate the associations between baseline covariates and the risk of cognitive impairment.

**Table S2. Association between nocturnal sleep duration and post-lunch napping**

| Nocturnal sleep duration (Hours) | Post-lunch napping (Minutes) | | | | $F$ or $\chi^2$ | $P$ | Bonferroni adjusted* |
|---|---|---|---|---|---|---|---|
| | 0 | 1~29 | 30~90 | >90 | | | |
| Mean（SD） | | | | | | | |
| | 6.28(0.03) | 6.35(0.05) | 6.53(0.04) | 6.89(0.06) | 19.7 | <0.001 | 1, 2, 3 < 4; 1, 2 < 3 |
| Frequency (%) | | | | | | | |
| ≤5 | 1134(32.8) | 350(28.8) | 472(25.4) | 168(20.8) | | | |
| 6~7 | 1378(39.8) | 539(44.3) | 818(44) | 305(37.8) | 100.68 | <0.001 | |
| ≥8 | 948(27.4) | 327(26.9) | 570(30.7) | 333(41.3) | | | |
| Total | 3460 | 1216 | 1860 | 806 | | | |

Kruskal-Wallis test with Bonferroni adjustment for post-hoc sub-group comparison was used for continues nocturnal sleep duration, and Pearson's $\chi^2$ test was used for categorical nocturnal sleep duration.

* 1: post-lunch napping = 0 minutes, 2: post-lunch napping=1~29 minutes, 3: post-lunch napping=30~90 minutes, 4: post-lunch napping > 90 minutes.
